# Dengue and zika seropositivity, burden, endemicity, and cocirculation antibodies in Nigeria

**DOI:** 10.1080/07853890.2023.2175903

**Published:** 2023-02-22

**Authors:** Peter Asaga Mac, Markos Tadele, Philomena E. Airiohuodion, Thilini Nisansala, Shaistha Zubair, Jude Aigohbahi, Chukwuma Anyaike, Raman Velayudha, Axel Kroeger, Marcus Panning

**Affiliations:** a Institute of Virologie, Universitatsklinikum, Freiburg, Freiburg, Germany; b Ethiopian Institute of Agricultural Research/EIAR, Addis Ababa, Ethiopia; c Special Programme for Research & Training in Tropical Diseases (TDR), World Health Organization, Switzerland; d Faculty of Veterinary Medicine, Universiti Malaysia Kelantan, Kelantan, Malaysia; e Faculty of Medicine, University of Maldives, Maldives; f Sanofi-Aventis Deutschland GmbH, Clinical trials, Frankfurt, Germany; g Federal Ministry of Health, Abuja, Nigeria; h World Health Organization, Geneva, Switzerland; i Centre for Medicine and Society, University of Freiburg, Freiburg, Germany

**Keywords:** Endemicity, chikungunya, dengue, Nigeria, seroprevalence, burden

## Abstract

**Introduction:**

Mosquito-borne infections are of global health concern because of their rapid spread and upsurge, which creates a risk for coinfections. DENV and ZIKV are transmitted by *Aedes aegypti* and *A. albopictus* and are prevalent in Nigeria and neighbouring countries. However, their seroprevalence, burden, hidden endemicity and possible cocirculation are poorly understood in Nigeria.

**Methods:**

We conducted a cross-sectional study of 871 participants from three regions of Nigeria. All serum samples were analysed using malaria RDT and the immunoblot molecular diagnostic assay recomLine Tropical Fever for the presence of arboviral antibody serological marker IgG (Mikrogen Diagnostik, Neuried, Germany) with DENV and ZIKV Nonstructural protein 1 (NS 1), DENV and ZIKV Equad (variant of the envelope protein with designated mutations to increase specificity), according to the manufacturer’s instructions.

**Results:**

The overall IgG antibody seropositivity against DENV-flavivirus was 44.7% (389/871); 95% CI (41.41–47.99), while ZIKV-flavivirus was 19.2% (167/871); 95% CI (0.16–0.21), and DENV-ZIKV-flavivirus cocirculation antibody seropositivity was 6.2%5 (54/871); 95% CI (0.6–0.7) in the three study regions of Nigeria. The study cohort presented similar clinical signs and symptoms of flaviviruses (DENV and ZIKV) in all three study regions.

**Conclusion:**

This study highlighted an unexpectedly high antibody seropositivity, burden, hidden endemicity, and regional spread of mono- and co-circulating flaviviruses (DENV and ZIKV) in Nigeria.

**Key messages**
- Dengue flavivirus sero-cross-reactivity drives antibody-dependent enhancement of ZIKV infection.
- Both viruses share common hosts (humans) and vectors (primarily Aedes aegypti), and are thus influenced by similar biological, ecological, and economic factors, resulting in epidemiological synergy.
- Additionally, the actual burden in epidemic and interepidemic periods is grossly or chronically unknown and underreported. Despite this trend and the potential public health threat, there are no reliable data, and little is known about these arboviral co-circulation infections.

## Background

Individuals living in arbovirus-endemic regions have reported complex arbovirus infections, including concurrent and simultaneous infections with dengue virus (DENV) and Zika virus (ZIKV), [1,2]. There is a high incidence of dengue fever in urban and semiurban environments, and it has spread to tropical areas, becoming one of the causes of death. Southeast Asia and Sub-Saharan Africa are the most affected regions, with high temperatures and poor sanitation [3]. Dengue fever is caused by four serotypes (DENV1-4) of flaviviruses [3]. An important symptom of this disease is fever, which is usually accompanied by severe body pain [3]. Zika virus is an emerging arthropod-borne virus belonging to the genus Flavivirus. The first human infection in Sub-Saharan Africa occurred in Nigeria in 1954 [4].

The emergence of dengue virus (DENV) and Zika virus (ZIKV) in chikungunya virus (CHIKV)-endemic regions has created intriguing but potentially alarming scenarios [5]. It has been postulated by a study conducted in Sri Lanka that for Zika and dengue viruses, prior infection with one virus modulates the severity of subsequent infection with the other virus [6].

This poses a major health concern, because of the high homology between these arboviruses, cross-reactivity of antibodies against flaviviruses such as DENV, ZIKV, yellow fever virus (YFV), tick-borne encephalitis virus (TBEV), and Japanese encephalitis virus (JEV) can occur, which may complicate the interpretation of serological results, coinfection and severe and foetal outcomes. There are several serodiagnostic tests for arboviral infections, including the enzyme-linked immunosorbent assay (ELISA), neutralization test (NT), immunofluorescence assay (IFA), and hemagglutination inhibition test. Dengue and Zika infections can be serologically diagnosed most reliably and specifically using PRNT [1,7]. However, PRNT is time consuming and requires a Biosafety Level 3 facility to handle live viruses. Comparatively, ELISA is simple and safe, but it is hindered by cross-reactivity among flaviviruses [1,2,4,5,8,9]. Both viruses share common hosts (humans) and vectors (primarily *Aedes aegypti*), and are thus influenced by similar biological, ecological, and economic factors [1,2,5,7,10], resulting in epidemiological synergy. Additionally, the actual burden in epidemic and interepidemic periods is grossly unknown and underreported [10]. Despite this trend and the potential public health threat, there are no reliable data, and little is known about the co-circulation of these arboviral infections. In the present study, we investigated the seropositivity, geographical spread, burden, and hidden endemicity of DENV and ZIKV, and their possible cocirculation (participants who were serologically positive for DENV and ZIKV during the sampling period or time) in three regions of Nigeria [1,4–6,10–12]. This will help in the characterization of the epidemiological patterns of these emerging viruses. These data will also assist clinicians and policymakers in designing and implementing effective control measures.

## Method

### Study design and site

**Figure 1.**
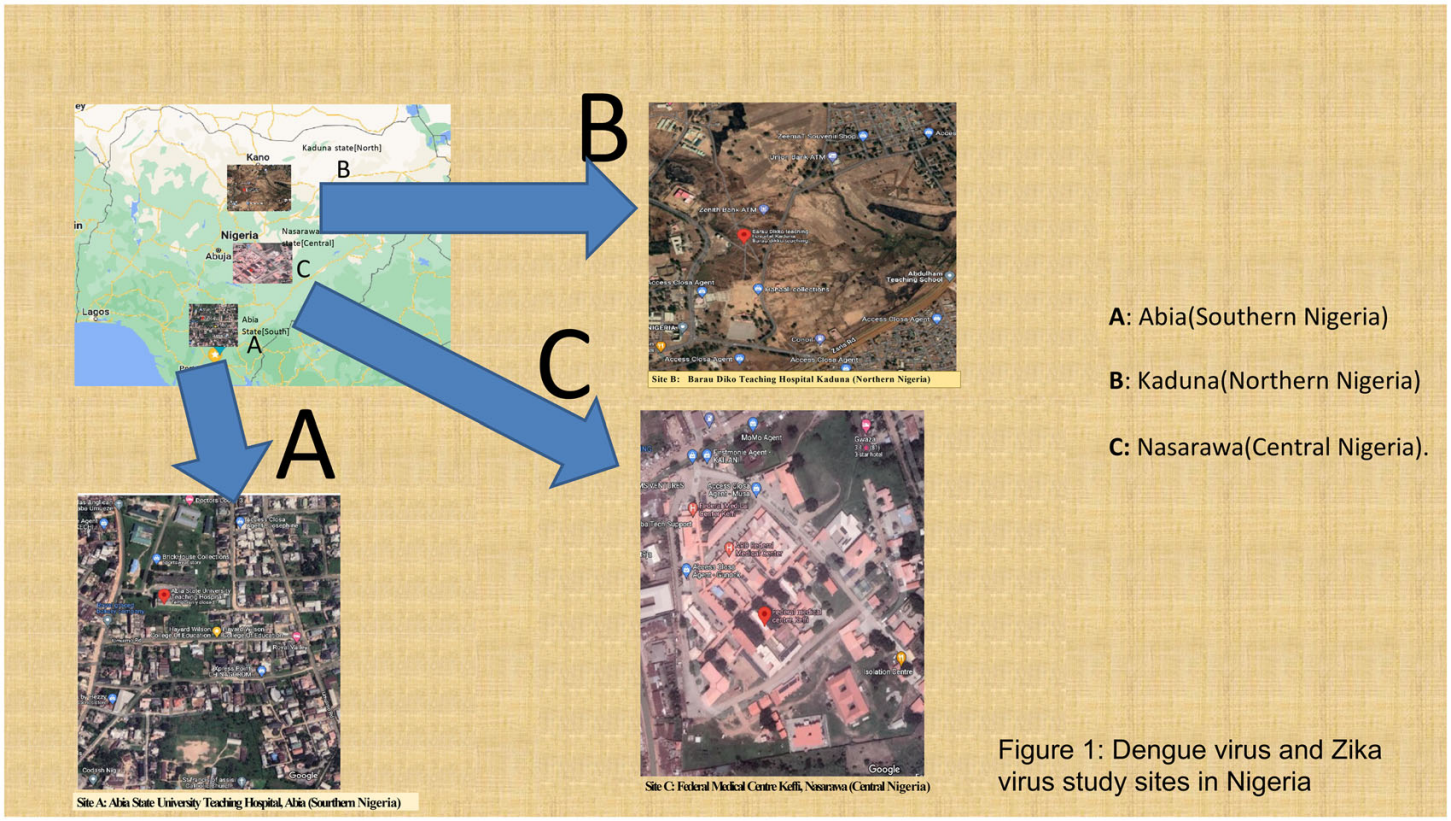
Dengue and Zika arboviral study sites in Nigeria.

This cross-sectional study was conducted in three university teaching hospitals located in three geographical regions of Nigeria: Nasarawa State in Central Nigeria, Abia State in Southern Nigeria, and Kaduna State in Northern Nigeria (Figure 1) [1]. The combined population of these regions is over 30 million. Forty-five percent of the population lives in urban areas (urban settlement in the context of the present study refers to high human population density and infrastructure of the built environment), 40% in rural areas (refers to an open countryside with population densities of less than 500 people per square mile or places with fewer than 1,500 people). and 15% in slums or informal settlements (informal settlements within urban cities with inadequate housing and squalid and miserable living conditions) [1]. The average annual temperatures in the southern regions range from 21 °C to 27 °C, whereas in the interior lowlands, temperatures are generally above 27 °C. The mean annual precipitation is 1,165.0 mm [1]. It rains throughout the year in most parts of southern and central Nigeria, with most rainfall occurring between April and October and minimal rainfall occurring between November and March in the northern region. The main occupations of people in the three regions are farming and trading in the southern and northern regions and mining and farming in the central region [1].

### Study population

The study population comprised outpatients, including pregnant women enrolled for antenatal care and patients presenting with illness at the rapid-access healthcare and antiretroviral (people living with AIDS) units of the hospitals between December 2020 and November 2021. These hospitals were purposefully selected to reflect the diversity of different cultures, religions, ethnicities, topographical and vegetation features, and different human activities in the three geographical regions. The inclusion criteria were all outpatients within an age range of 0 months to 80 years who agreed to participate in the study and signed the consent form, including children whose parents or guardians gave consent, while exclusion criteria were participants who were already undergoing treatment, those who refused to sign the consent form, and seriously ill patients who were hospitalized.

A clinical research form (structured questionnaire) was used to obtain information that included questions on demographics, medical history, vital signs and symptoms, clinical evaluation, data on hospitalization, and a summary form. All study subjects were screened for malaria-, DENV-, and ZIKV-related symptoms (fever, headaches, rashes, joint pain, conjunctivitis, and muscular pain) (Table 1). Detailed protocol information was made available and fully explained to the participants in English and their respective local languages before enrolment. The study participants signed an informed consent form after enrollment. Participants who could not read and write were asked to verbally consent and then to thumbprint, indicating that they were willing to participate in the study.

**Table 1.** Signs and symptoms presented by Zika or dengue monoinfected patients.

	Mono-infection (% sign & symptoms)	
Sign and Symptoms	Anti-Zika positive (*N* = 167)	Anti-dengue positive (*N* = 389)
Headaches	28.1% (47/167)	91.0% (354/389)
Exanthema	12.6% (21/167)	28.0% (109/389)
Fever	29.3% (49/167)	97.4% (379/389)
Abdominal pain	60.5% (101/167)	29.5% (115/389)
Diarrhoea	49.1% (82/167)	26.9% (105/389)
Myalgia	23.4% (39/167)	24.7% (96/389)
Vomiting	53.3% (89/167)	30.8% (120/389)
Generalised body pains	30.5% (51/167)	73.8% (287/389)
Arthralgia	26.9% (45/167)	46.0% (179/389)
Edema	35.3% (59/167)	0.0% (0/389)
Haemorrhagic manifestation	0.0% (0/167)	3.6% (14/389)
Retro-orbital pain	7.2% (12/167)	6.4% (25/389)
Nausea	3.0% (5/167)	3.1% (12/389)
Non-purulent conjunctivitis	1.2% (2/167)	1.3% (5/389)
Leuckopenia	50.9% (85/167)	30.8% (120/389)

We collected 871 samples from study participants in three regions by employing a simple random sampling method between December 2020 and November 2021. Of these, 262 samples were from outpatients, 499 were from HIV-positive patients, and 110 were taken from blood banks. The sample size calculation (based on a 40% expected proportion of DENV and ZIKV in a total population of five hundred thousand patients with a confidence interval of 95% and a p value of 0.05) [1,13] showed a minimum sample size of 384 serum samples, which we increased to 871 samples to be able to analyse subgroups according to regions.

Venous blood (5 mL) was collected from all study participants by a local clinical diagnostic laboratory technician (located in the hospital, who collected patient blood samples daily). The serum was extracted and screened at the study site for malaria parasites using an RDT kit (SD BIOLINE Malaria Differential P.f/Pan Ag RDT (HRP II + pLDH, Abbott, USA), according to the manufacturer’s instructions. The samples were shipped on dry ice to the Institute of Virology (Freiburg, Germany). The samples were stored at −20 °C for DENV and ZIKV antibody analysis. DENV and ZIKV, analyses were performed using the Immunoblot molecular diagnostic assay recomLine Tropical Fever for the presence of arboviral antibody serological marker IgG (Mikrogen Diagnostik, Neuried, Germany) with DENV and ZIKV Nonstructural protein 1 (NS 1), DENV and ZIKV Equad (Variant of the envelope protein with designated mutations to increase specificity), according to the manufacturer’s instructions [14]. This test is highly specific because of the targeted mutations.

### Statistical tests

Statistical analysis was performed using SPSS version 28, IBM, USA. Descriptive statistics were employed for the analysis of the results, and we tested for associations between demographics and DENV and ZIKV antibody seropositivity using regression. The results were deemed statistically significant at a p-value ≤0.05, and odds ratios (OR) at a confidence interval (CI) of 95%.

### Ethics statement

The study protocol was reviewed and approved by the local ethics committee on human research at the Universitatsklinikum, Freiburg [No. 140/19], and the local ethics committee on human research at the Tertiary Hospitals and National Ethics Committee on Human Research of Nigeria [No KF/REC/02/21].

## Results

### Flaviviruses serology

Due to mild and nonspecific symptoms, serological tests are essential in epidemiological studies. Interpretation of these serological tests (immunoblot assay) may be complicated by cross-reactive antibodies between flaviviruses and alphaviruses. Therefore, the test interpretation for the current study may be presented as flavivirus seropositive for DENV and ZIKV.

### The study population

A total of 871 participants were recruited from three geographical areas of Nigeria:17.5% (152/871) from southern Nigeria (Abia), 34.4% (300/871) from northern Nigeria (Kaduna), and 48.1% (419/871) from central Nigeria (Nasarawa). The study population had an age range of 0 months to 80 years, with a mean age of 36.5 years. Female participants comprised 71% (619/871) of the study population, while male participants constituted 29% (252/871). Approximately 28.1% (14/167) of the study participants who showed signs and symptoms of antibody seropositivity against ZIKV had headaches, whereas 91.0% of those with headaches had signs and symptoms of DENV seropositive antibodies. ZIKV (fever, 29.3%) DENV (fever, 97.4%), ZIKV (Exanthema, 12.6%), DENV (Exanthema, 28.0%), ZIKV (Abdominal pain, 60.5%), DENV (Abdominal pain, 29.5%). The study cohort exhibited varied but similar clinical signs and symptoms of flaviviruses across all three study regions (Table 1). The overall IgG antibody seropositivity against flavivirus-DENV was 44.7% (389/871); 95% CI (41.41–47.99), while ZIKV-flavivirus was 19.2% (167/871); 95% CI (0.16–0.21), whereas the antibody seropositivity against one or more flaviviruses (either DENV or ZIKV participants who were or who showed serological evidence against DENV and ZIKV at sampling period or time) cocirculation was 6.2%5 (54/871); 95% CI (0.6–0.7) (Table 2).

**Table 2.** Sociodemographic characteristics and seropositivity of dengue, Zika and dengue-Zika cocirculation arboviral infection.

	Dengue virus						Zika virus						Dengue-Zika cocirculation					
Region	Negative	Positive	Total Examined (n)	95%CI	OR	*p*-value	Negative	Positive	Total Examined (n)	95%CI	OR	*p*-value	Negative	Positive	Total Examined (n)	95%CI	OR	*p*-value
Southern Nigeria	94 (61.8%)	58 (38.2%)	152 (100%)	0.53–1.08	0.8	0.01	119 (78.3%)	33 (21.7%)	152 (100%)	0.76–1.78	1.2	0.40	139 (91.4%)	13 (8.6%)	152 (100%)	0.75–2.66	1.4	0.21
Northern Nigeria	189 (63.0%)	111 (37.0%)	300 (100%)	0.55–0.95	0.7		245 (81.7%)	55 (18.3%)	300 (100%)	0.67–1.32	1.0		287 (95.7%)	13 (4.3%)	300 (100%)	0.36–1.27	0.7	
Central Nigeria	199 (47.5%)	220 (52.5%)	419 (100%)	1.08–1.73	1.4		340 (81.1%)	79 (18.9%)	419 (100%)	0.72–1.31	1.0		391 (93.3%)	28 (6.7%)	419 (100%)	0.67–1.73	1.1	
**Sex**																		
Male	150 (59.5%)	102 (40.5%)	252 (100%)	0.63–1.12	0.8	0.20	200 (79.4%)	52 (20.6%)	252 (100%)	0.77–1.55	1.1	0.60	240 (93.2%)	12 (4.8%)	252 (100%)	0.39–1.43	0.8	0.65
Female	332 (53.6%)	287 (46.4%)	619 (100%)	0.87–1.31	1.1		504 (81.4%)	115 (18.6%)	619 (100%)	0.73–1.25	1.0		577 (93.2%)	42 (6.8%)	619 (100%)	0.72–1.67	1.1	
**Domicile**																		
Urban	313 (61.7%)	194 (38.3%)	507 (100%)	0.61–0.96	0.8	0.02	412 (81.3%)	95 (18.7%)	507 (100%)	0.73–1.28	1.0	0.84	483 (95.3%)	24 (4.7%)	507 (100%)	0.45–1.23	0.8	0.11
Rural	122 (47.3%)	136 (52.7%)	258 (100%)	1.04–1.82	1.4		207 (80.2%)	51 (19.8%)	258 (100%)	0.73–1.47	1.0		239 (92.6%)	19 (7.4%)	258 (100%)	0.69–2.06	1.2	
Slum	47 (44.3%)	59 (55.7%)	106 (100%)	1.03–2.33	1.6		85 (80.2%)	21 (19.8%)	106 (100%)	0.62–1.72	1.0		95 (89.6%)	11 (10.4%)	106 (100%)	0.88–3.46	1.7	
**Marital status**																		
Married	351 (54.3%)	296 (45.7%)	647 (100%)	0.85–1.28	1.0	0.20	527 (81.5%)	120 (18.5%)	647 (100%)	0.73–1.27	1.0	0.42	620 (95.8%)	27 (4.2%)	647 (100%)	0.41–1.05	0.7	0.00
Single	126 (57.8%)	92 (42.2%)	218 (100%)	0.67–1.22	1.0		171 (78.4%)	47 (21.6%)	218 (100%)	0.80–1.66	1.2		192 (88.1%)	26 (11.9%)	218 (100%)	1.25–3.35	2.0	
Divorced	5 (83.3%)	1 (16.7%)	6 (100%)	0.03–2.13	0.2		6 (100%)	0 (0%)	6 (100%)	0.02–5.77	0.3		5 (83.3%)	1 (16.7%)	6 (100%)	0.34–26.3	3.0	
**HIV status**																		
HIV-positive Pregnant	118 (42.9%)	157 (57.1%)	275 (100%)	1.25–2.16	1.7	0.00	217 (78.9%)	58 (21.1%)	275 (100%)	0.80–1.57	1.2	0.17	262 (95.3%)	13 (4.7%)	275 (100%)	0.40–1.39	0.8	0.00
HIV-positive nonpregnant participants	130 (58.0%)	94 (42.0%)	224 (100%)	0.66–1.20	0.9		199 (88.8%)	25 (11.2%)	224 (100%)	0.33–0.82	0.5		187 (83.5%)	37 (6.5%)	224 (100%)	1.91–4.68	3.0	
HIV-negative participants	234 (62.9%)	138 (37.1%)	372 (100%)	0.56–0.93	0.7		288 (77.4%)	84 (22.6%)	372 (100%)	0.91–1.65	1.3		368 (98.9%)	4 (1.1%)	372 (100%)	0.05–0.45	0.2	
**Malaria status**																		
Malaria positive	344 (54.5%)	287 (45.5%)	631 (100%)	0.84–1.27	1.0	0.75	511 (81.0%)	120 (19.0%)	631 (100%)	0.76–1.28	1.0	0.88	597 (94.6%)	34 (5.4%)	631 (100%)	0.55–1.34	1.0	0.24
Malaria negative	136 (56.7%)	104 (43.3%)	240 (100%)	0.71–1.26	1.0		193 (80.4%)	47 (19.6%)	240 (100%)	0.71–1.47	1.0		220 (91.7%)	20 (8.3%)	240 (100%)	0.80–2.34	1.4	
**Blood product source**																		
Outpatient serum	457 (60.1%)	304 (39.9%)	761 (100%)	0.67–1.00	0.9	0.00	663 (87.1%)	98 (13.9%)	761 (100%)	0.14–0.23	0.1	0.00	738 (97.0%)	23 (3.0%)	761 (100%)	0.02–0.06	0.0	0.00
Blood Bank serum	25 (22.7%)	85 (77.3%)	110 (100%)	2.64–6.71	4.2		41 (37.3%)	69 (62.7%)	110 (100%)	1.38–3.13	2.1		79 (71.8%)	31 (28.2%)	110 (100%)	0.31–0.75	0.5	
**Grand Total Examined (N)**	482 (55.3%)	389 (44.7%)	871 (100%)	0.43–0.45			704 (80.8%)	167 (19.2%)	871 (100%)	0.18–0.20			817 (80.8%)	54 (6.2%)	871 (100%)	0.5–0.7		

### Sociodemographic characteristics and seropositivity of dengue and zika arboviral infection in the study population

#### Regions

A subgroup analysis of the various regions showed significant antibody seropositivity against DENV-flavivirus in the central region [52.5% (220/419); 95% CI (1.08–1.73); OR = 1.4, *p* = 0.00], whereas ZIKV-flavivirus [21.7% (33/152); 95% CI (0.76–1.78); OR = 1.2, *p* = 0.40] and either DENV-ZKIV-flavivirus [8.6% (13/152); 95% CI (0.75–2.66); OR = 1.2, *p* = 0.21] antibody seropositivity cocirculation was higher in the southern region. Antibody seropositivity against DENV-flavivirus (37.0% (11/300); 95% CI (0.55–0.95) and ZIKV-flavivirus (18.3% (55/300); 95% CI (0.67–1.23) was much lower in the northern region. Similarly, DENV-ZIKV cocirculation was also much lower in the northern region. The odds of DENV-flavivirus were 1.4 times higher in the central region, and the odds of ZIKV and DENV-ZIKV (flaviviruses) cocirculation were higher in the southern region (Table 2).

### Sex-specific seropositivity of dengue and zika arboviral infection

There was considerable DENV-flavivirus antibody seropositivity among the female participants [46.4% (287/619); 95% CI (0.87–1.31); OR = 1.1, *p* = 0.20], and lower DENV-flavivirus seropositive antibodies in male individuals (40.5% (102/252); 95 CI (0.63–1.12). Antibody seropositivity against ZIKV flavivirus [20.6% (52/252); 95% CI (0.77–1.55); OR-1.1, *p* = 0.60] was more pronounced among male subjects and less elevated in the female participants (18.6% (115/619); 0.73–1.25). DENV-ZIKV-flavivirus cocirculation antibody seropositivity were highest among the female participants (6.8% (42/619); 95% CI (0.72–1.67) than the male participants (4.3% (12/252); 0.39–1.42) (Table 2).

### Place-specific seropositivity of dengue and zika

The highest antibody seropositivity against DENV-flavivirus [55.7% (59/106); 95% CI (1.03–2.33); OR = 1.6, *p* = 0.02] and DENV-ZIKV-flavivirus cocirculation [10.4% (11/106); 95% CI (0.88–3.46); OR = 1.7, *p* = 0.00] was observed among slum participants, whereas the lowest seropositive antibodies against DENV-flavivirus (38.3% (194/507); 95% CI (0.61–0.96), *p* = 0.00), ZIKV-flavivirus (18.7% (95/507), and DENV-ZIKV-flavivirus (4.7%(24/507) were more prevalent among the urban participants. The odds of DENV-flavivirus seropositivity and DENV-ZIKV-flavivirus cocirculation were higher in the slum group than in the other groups (Table 2).

### Marital status seropositivity of dengue and zika arboviral infection

Antibody seropositivity against DENV-flavivirus was more evident among married participants [45.7% (296/647); 95% CI (0.85–1.28); OR = 1.0, *p* = 0.20], followed by single individuals (42.2% (92/218). ZIKV flavivirus antibody was the highest among single participants (21.6% (47/218); 95% CI (0.80–1.66); OR = 1.2, *p* = 0.42), and interestingly, divorced individuals had the most seropositive antibodies against DENV-ZIKV flavivirus cocirculation (16.7% (1/6); 95% CI (0.34–26.3); OR = 3.0, *p* = 0.00). However, the odds of DENV-ZIKV-flavivirus were 3.0 times higher among the divorced group than in the other groups (Table 2).

### HIV status- specific antibody seropositivity of dengue and zika in the study population

Flavivirus antibody seropositivity against DENV was significantly higher among HIV-positive pregnant participants [57.1% (157/275); 95% CI (1.25–2.16); OR = 1.7, *p* = 0.00], while flavivirus antibody seropositivity against ZIKV (21.1% (58/275); 95% CI (0.80–1.57); OR = 1.3, *p* = 0.17) was slightly lower in HIV-positive pregnant women compared to flavivirus-DENV antibody seropositivity among HIV-negative participants [22.6% (84/372); 95% CI (0.91–1.65); OR = 1.3, *p* = 0.17]. However, the odds of flavivirus antibody seropositivity against DENV-ZIKV cocirculation was 3.0 times higher among HIV-positive non-pregnant participants than among the other HIV status groups. The results were considered to be statistically significant (Table 2).

### Malaria status seropositivity of dengue and zika

Remarkable flavivirus antibody seropositivity against DENV and ZIKV was observed among malaria-positive patients [45.5% (287/631); 95% CI (0.84–1.27); OR = 1.0, *p* = 0.75] and malaria-negative patients (19.6% (47/240); 95% CI (0.71–1.47); OR = 1.0, *p* = 0.88), while much lower DENV-ZIKV flavivirus antibody seropositivity was observed among malaria-positive patients (5.4%) (34/631) compared to malaria-negative patients (8.3% (20/240) (Table 2).

### Outpatients and blood bank-specific seropositivity of dengue and zika

Flavivirus seropositive antibodies against DENV [77.3% (85/110); 95% CI (2.64–4.71); OR = 4.2, *p* = 0.00] and ZIKV [62.7% (69/110); 95% CI (1.38–3.13); OR = 2.1, *p* = 0.00] were significantly higher in serum samples from the blood bank. DENV-flavivirus (39.9% (304/761); 95% CI (0.67–1.00), OR = 0.9, *p* = 0.00) and ZIKV-flavivirus (13.9% (98/761); 95% CI (0.14–0.30), *p* = 0.00) seropositive antibodies were much lower than those in samples from outpatients, while DENV-ZIKV flavivirus antibody seropositivity was also significantly elevated in sera from blood banks (28.2% (31/110) compared to sera from outpatient blood products (3.0 (23/761]) . There was a statistically significant difference between the blood product and flavivirus antibody seropositivity in the study population (Table 2).

### Age-specific antibody seropositivity of dengue, zika, and dengue-Zika cocirculation

There were unexpectedly high flavivirus-DENV seropositive antibodies among the 0- to 9-year-old group (14 days and 6 months old infants) [66.7% (2/3); 95% CI (0.61–0.71); *p* = 0.04], which was statistically significant, whereas flavivirus seropositive antibodies against ZIKV were highly prevalent among the 50- to 59-year-old group [32.9% (27/82); 95% CI (0.32–0.34); *p* = 0.06]. The highest flavivirus (DENV-ZIKV) cocirculation antibody seropositivity rate was observed in the 80-year-old group [16.7% (1/6); 95% CI (12.7–20.7); *p* = 0.01]. There was a statistically significant difference between flavivirus cocirculation and the various age groups (Table 3).

**Table 3.** Age-specific seropositivity of dengue, Zika and dengue-Zika cocirculation in the study population.

	Dengue virus					Zika Virus					DENVZIKV Cocirculation				
Age group	Negative	Positive	Total Examined (n)	95%CI		Negative	Positive	Total Examined (n)	95%CI		Negative	Positive	Total Examined (n)	95%CI	
0–9	1 (33.3%)	2 (66.7%)	3 (100%)	0.61–0.72	*p = 0.04*	3 (100%)	3 (0.0%)	3 (100%)	−5.7–5.7	*p = 0.06*	3 (100%)	0 (0.0%)	3 (100%)	−5.7–5.7	*p = 0.01*
10–19	34 (66.7%)	17 (33.3%)	51 (100%)	0.32–0.35		40 (78.4%)	11 (21.6%)	5 (100%)	0.10–0.32		50 (98.0%)	1 (2.0%)	51 (100%	0.73–3.46	
20–29	120 (61.2%)	76 (38.8%)	196 (100%)	0.38–0.40		167 (85.2%)	29 (14.8%)	196 (100%)	0.14–0.16		188 (95.9%)	8 (4.1%)	196 (100%)	3.4–4.8	
30–39	173 (54.6%)	144 (45.4%)	317 (100%)	0.45–0.46		253 (79.8%)	64 (20.2%)	317 (100%)	0.20–0.21		294 (92.7%)	23 (7.3%)	317 (100%)	6.8–7.9	
40–49	81 (44.8%)	100 (55.2%)	181 (100%)	0.54–0.56		154 (85.1%)	27 (14.9%)	181 (100%)	0.14–0.16		167 (97.3%)	14 (7.7%)	181 (100%)	6.9–8.4	
50–59	47 (57.3%)	35 (42.7%)	82 (100%)	0.42–0.44		55 (67.1%)	27 (32.9%)	82 (100%)	0.32–0.34		75 (91.5%)	7 (8.5%)	82 (100%)	7.4–9.6	
60–69	15 (55.6%)	12 (44.4%)	27 (100%)	0.43–0.46		21 (77.8%)	6 (22.2%)	27 (100%)	0.20–0.24		27 (100%)	0 (0.0%)	27 (100%)	−1.9–1.9	
70–79	6 (75.0%)	2 (25.0%)	8 (100%)	0.22–0.28		6 (75.0%)	2 (25.0%)	8 (100%)	0.22–0.28		8 (100%)	0 (0.0%)	89100%)	−1.0–1.0	
80+	5 (83.3%)	1 (16.7%)	6 (100%)	12.7–20.7		5 (83.3%)	1 (16.7%)	6 (100%)	12.7–20.7		5 (83.3%)	1 (16.7%)	6 (100%)	12.7–20.7	
Total	482 (55.3%)	389 (44.7%)	871 (100%)	0.41–0.47		704 (80.8%)	167 (19.2%)	871 (100%)	0.16–0.21		817 (93.8%)	54 (6.2%)	871 (100%)	0.6–0.7	

## Discussion

The findings of our study are significant given that the transmission and geographic spread of DENV and ZIKV (flaviviruses) are not well documented in Nigeria. In the absence of antibodies against other flaviviruses or alphaviruses, seropositivity for the two arboviruses was detected. Consequently, circulation of each of the two targeted arboviruses was confirmed. The presence of IgG antibodies indicated that the participants were previously infected with DENV and ZIKV, as determined by immunoblot assay serology. IgG antibodies can be detected for years or even a lifetime [11]; therefore, in the current study, we could not determine when the study participants were infected with the two flaviviruses.

The cocirculation of flaviruses in Nigeria poses a significant threat to public health, especially dengue and Zika, which remain undetected in the face of emerging climatic changes in the three regions as a result of communal clashes in the central and northern regions of Nigeria and an increase in population density in the southern regions. The study cohort presented similar clinical signs and symptoms of flaviviruses (DENV and ZIKV) in all three study regions. The antibody seropositivity against both flaviviruses [(DENV (44.7%) and ZIKV) (19.2%) was significantly higher in the three profiled regions. In addition, 45.5%, 19.0%, and 5.4% of DENV seropositive, ZIKV seropositive, and DENV-ZIKV seropositive patients, respectively, were also positive for the malaria antigen. However, much lower seroprevalences have been reported elsewhere in Nigeria, most recently for dengue (34.7%, 9.4%, 10.1%, 18.0%) [8,9,15–25] and ZIKV (2.0%,12%) [6,7,10–12,26]. This confluence of heterogeneous findings could be explained by the common vector of transmission (*Aedes* mosquito) [1–7,10–12,26–28] occurring in the three geographic areas simultaneously. The result might have also been influenced or shaped by DENV and ZIKV antibody cross-reactivity, past exposure to the arboviral vaccine (possibly yellow fever vaccine), and long-term exposure immunity. In this study, recombinant flavivirus-DENV and ZIKV antigens were used in the immunoblot assay, and the antibodies were considered specific to DENV and ZIKV. It is likely that the different levels of endemicity (antibody seropositivity) against the two arboviruses in the three regions also resulted from different vector densities due to different vegetation types across the three regions, human population indices, climate changes (increasing reproductive activities and shortening the extrinsic cycle of arboviruses in vectors), vector adaptations, variations in temperature and humidity, and flooding (which favours the emergence and survival of arboviruses), which shape habitats and microclimates. Unplanned urbanization in the three regions favours mosquito-borne vector transmission dynamics [8,26]. Additionally, because of the limited testing capabilities of regional health systems to accurately diagnose arboviral infections and differentiate them from other febrile illnesses, this disease remains a hidden burden in various demographic groups as it is usually undetected by health services. The high antibody seropositivity results across the three regions were also influenced by the sampling period and molecular diagnostic method employed. Although our results are consistent with those of other studies, diagnostic methods as well as less extensive seroprevalence studies have been conducted across Nigeria, West Africa, and elsewhere [1–7,10–12,20,27,29]. To the best of our knowledge, no known DENV-ZIKV cocirculation study has been conducted in Nigeria. The current study, which is the first in the country, revealed a definitive seropositivity rate of 6.2% among the study participants in the three regions. Elsewhere, a seropositivity of 6.3% [1] was reported in the Columbia-Venezuela border, 2.6% [13,30–32] in Brazil and 1.7% [5] in Nicaragua. We also speculate from our findings in the three regions that antibody seropositivity against cocirculation is higher than currently perceived due to the rapid introduction of ZIKV in countries that are endemic for DENV [6,7,10–12,26].

Although DENV and ZIKV antibody seroprevalence seemed to increase with age in the present study, unexpectedly high seropositive antibodies against DENV were observed in children. This could be attributed to the asymptomatic signs (which could possibly be explained by maternal seropositivity against DENV and ZIKV. They were 14 days and 6 months old infants) and hidden endemicity and burden of DENV and ZIKV in Nigeria. We found that DENV and ZIKV antibodies were more prevalent in older age groups in the 3 regions [1,17]. Several factors can contribute to this, including past infections (exposure over time), immunosenescence in old age, long-standing immunity to arboviruses, and increased vector exposure in relation to activities close to mosquito breeding habitats. Older people are also more likely to be bitten by *Aedes* mosquitoes because they sit in unscreened areas for long periods each day (day feeding activities of *Aedes aegypti*).

In the present study, seropositive anti-DENV and anti-ZIKV were equally detected in all participants from the three settlement types (slum, rural, and urban, although slightly more in slums). This phenomenon may be attributed to several reasons, including rural–urban migration resulting from political conflict or exhaustion, especially in northern and central Nigeria, and travel and commercial activities that lead to overcrowding [33–36] resulting in outbreaks of unknown DENV and ZIKV in these urban and slum areas [18,19,28]. The risk of vector exposure also increases in urban and slum areas where mosquito breeding habitats are prevalent, including refuse disposal dumpsites, unhygienic sewage and drainage systems, stagnant water in tyres, and tin containers, which serve as suitable habitats for *Aedes* species [1,4–8,10].

The antibody seropositivity against DEN and ZIKV was high, but almost the same across HIV-positive and HIV-negative individuals. These findings seem ambiguous, but the most valid explanation could be that most of the study participants were women, which could be attributed to the health-seeking behaviours of women and the strict adherence to antiretroviral medication among HIV-positive individuals and routine antenatal care during pregnancy [11], as most of the study participants from the three regions were recruited from the antiretroviral and antenatal units of tertiary healthcare centres.

DENV-seropositive antibodies were notably higher in married participants than in single and divorced individuals. However, seropositive antibodies against ZIKV and DENV-ZIKV cocirculation were higher among single and divorced participants. This is not far from the fact that ZIKV is transmitted sexually [1–5,8,12,26–28]. There is also the possibility of overcrowding of families in major urban cities in the three regions, leading to a high burden on social services [8,9,15–25], which in turn contributes to high rates of DENV and ZIKV arboviral infection transmissibility.

The reason for the marked DENV and ZIKV seropositive antibodies among malaria-positive and malaria-negative participants remains unclear. It is unknown in the present study whether the presence of DENV and ZIKV seropositive antibodies increases or reactivates malaria infection in the presence of the malaria parasite [15,16,29,37]. However, several studies have reported that concomitant or co-circulation of malaria and arboviruses may increase seroprevalence rates, especially in tropical and subtropical regions [4,19,20].

Serum samples from blood banks showed the most seropositive antibodies against DENV, ZIKV, and DENV-ZIKV cocirculating antibodies. This could be attributed to blood donation by asymptomatic individuals and the failure, lack, and inability of regional health services or systems to diagnose and distinguish between malaria (most times, they screen for malaria but not arboviral infections) and other febrile illnesses.

This study had several significant limitations. The cross-reactivity of IgG antibodies between flaviviruses and other alphaviruses is well established and a confounding factor for serological studies investigating the seropositivity of arboviruses. All serum samples that were seropositive for both DENV and ZIKV, or both, were classified as flavivirus-positive. Because of the large sample size, it was impractical to conduct additional testing using techniques such as the plaque reduction neutralization test (PRNT), other sero-neutralization tests, and PCR. The current study was conducted in tertiary hospitals in three regions of Nigeria; therefore, it may not be representative of true dengue and Zika seropositivity. Additionally, we did not perform plaque reduction neutralization tests (PRNT) or PCR to confirm DENV and ZIKV, and there is a likelihood of false negatives or positives due to cross-reactivity and arboviral vaccines. There were more females than males, which may have led to bias and confounding variables, as well as age.

## Conclusion

This study revealed significant antibody seropositivity, hidden endemicity, and burden of DENV, ZIKV, and DENV-ZIKV cocirculation in Nigerian communities. The results may also have been influenced or shaped by DENV and ZIKV antibody cross-reactivity, past exposure to the arboviral vaccine (possibly yellow fever vaccine), long-term exposure immunity, different vector densities due to different vegetation types across the three regions, human population indices or anthropogenic activities, climate change, vector adaptations, variations in temperature and humidity, and flooding, which shape habitats and microclimates. Unplanned urbanization in the three regions which favours mosquito-borne vector transmission dynamics

Therefore, differential diagnosis should be performed in patients with acute febrile syndrome, and screening of blood donors for arboviral infections will assist clinicians and policymakers in designing, generating data, and implementing effective control measures.

## Data Availability

All data are contained in the manuscript.
